# Ancient DNA Indicates Farmers, not Just Farming, Spread West

**DOI:** 10.1371/journal.pbio.1000535

**Published:** 2010-11-09

**Authors:** Richard Robinson

**Affiliations:** Freelance Science Writer, Sherborn, Massachusetts, United States of America

**Figure:**
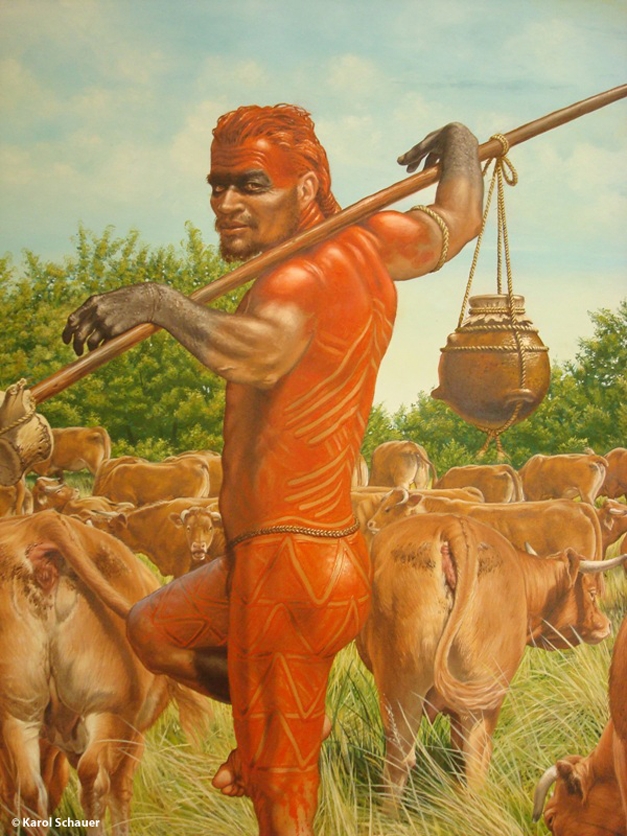
First farmer of the Linear Pottery Culture in Neolithic Central Europe. Illustration: Karol Schauer, State Museum of Prehistory in Halle (Saale), Saxony-Anhalt, Germany.

How did farming spread to Europe, from its origins in the Middle East? Did indigenous European hunter–gatherers adopt more settled ways while largely maintaining the integrity of their population group, or did farmers from the east spread into Europe, supplanting or mixing with the indigenous population? That question remains controversial among archaeologists, anthropologists, and population geneticists, with conflicting evidence supporting each argument. In a new study in *PLoS Biology*, Wolfgang Haak and colleagues make a strong case for the latter view, through their analysis of DNA drawn from an ancient graveyard in modern-day Germany.

Until recently, the study of the genetics of ancient peoples relied solely on analysis of the DNA of modern populations, using their similarities and differences to make inferences about migrations and mixtures. But DNA can be preserved in ancient bones, and advances in techniques of extraction and analysis have let researchers probe those samples. In this study, the authors drew samples from 22 individuals buried in a graveyard in near the village of Derenberg in the German state of Saxony-Anhalt, about 100 miles southwest of Berlin. The graves date from about 7,100 years ago, and the individuals in the graves were farmers from the Early Neolithic “Linear Pottery Culture” (abbreviated LBK), which ranged from modern-day Ukraine to France. The present study expands on the authors’ previous work on samples over a broader geographic area. The exclusive focus here on individuals from a single site provided them an opportunity to more rigorously characterize the genetic structure of a single population.

Mitochondrial DNA is the sample of choice for studies in such ancient samples because it provides many more copies for analysis than nuclear DNA. While it is possible to sequence and compare the entire mitochondrial genome, it is easier, and more common, to focus on and compare “haplotypes”—defining DNA segments. The existence of the same haplotype in individuals from two regions suggest they share a common ancestor.

The authors compared mitochondrial haplotypes from the LBK farmers to modern individuals from 36 regions across Eurasia. Nine modern population pools had mitochondrial DNA that was closely affiliated with the LBK population. Three, from central Europe, were no surprise, since the LBK culture is believed to have arisen there. At least one other, from England, may represent a statistical anomaly rather than a true link to historical origins. But four others link the LBK culture to populations in modern-day Turkey, Syria, Iraq, and other regions in the Near East.

The authors checked their results in a variety of ways using a range of models, all with the same result. They also analyzed Y chromosome DNA, and, while the data were not as conclusive, these results also supported the mitochondrial analysis. Together, they appear to indicate that ancient movement of peoples, not just their cultures, brought agriculture to Europe. The indigenous hunter–gatherer population, which had itself colonized Europe following the retreat of the last glaciers, likely did not die out as the farmers moved in; instead, they mingled with them, creating the mixed DNA found in the individuals from the graveyard.

The data even suggest a route of migration from the East. If the degree of genetic dissimilarity between groups is taken as a marker of time since they diverged, the migrants appear to have traveled west across Anatolia and the Balkans, then northward along the Danube via the fertile Carpathian Basin into central Europe, following, during their centuries-long migration, both the geography and the fertility of the land.

The authors acknowledge that theirs is unlikely to be the last word on this subject; the puzzle of human movements over thousands of years is too complex to be neatly solved with one study. Instead, more data from other ancient sites, both in Europe and the Near East, will be needed to better understand how patterns of migration aided the spread of agriculture from east to west.

**Haak W, Balanovsky O, Sanchez JJ, Koshel S, Zaporozhchenko V, et al. (2010) Ancient DNA from European Early Neolithic Farmers Reveals Their Near Eastern Affinities. doi:10.1371/journal.pbio.1000536**

